# Diethyl 2,2′-[({2-chloro-5-[(2-eth­oxy-2-oxoeth­yl)(2-methyl­indolin-1-yl)carbamo­yl]phen­yl}sulfon­yl)aza­nedi­yl]di­acetate

**DOI:** 10.1107/S2414314623006995

**Published:** 2023-08-24

**Authors:** Youssef Ramli, Wedad Al Garadi, Mohamed El Hafi, Elghayati Lhoussaine, El Mokhtar Essassi, Abdulsalam Alsubari, Joel T. Mague

**Affiliations:** a Laboratory of Medicinal Chemistry, Drug Sciences Research Center, Faculty of Medicine and Pharmacy, Mohammed V University in Rabat, Rabat, Morocco; b Mohammed VI Center for Research and Innovation (CM6), Rabat 10000, Morocco; c Laboratoire de Chimie Organique Heterocyclique, Faculté des Sciences, Université Mohammed V Rabat, Morocco; d Laboratory of Medicinal Chemistry, Faculty of Clinical Pharmacy, 21 September University, Yemen; e Department of Chemistry, Tulane University, New Orleans, LA 70118, USA; Sunway University, Malaysia

**Keywords:** crystal structure, hydrogen bond, C—H⋯π(ring) inter­action, indapamide, indole, sulfamate ester

## Abstract

The majority of the title mol­ecule, C_28_H_34_ClN_3_O_9_S, is disordered over two closely spaced sets of sites; the site occupancy of the major component = 0.542 (3). The conformation of each component is approximately U-shaped with the chloro­benzene ring forming the base and the indolinyl and sulfamoyl groups the sides; an intra­molecular C—H⋯Cl hydrogen bond possibly contributes to the stabilization of the conformation. In the crystal, a corrugated layer structure parallel to the *ab* plane is formed by C—H⋯O and C—H⋯Cl hydrogen bonds together with C—H⋯π(ring) inter­actions.

## Abstract 2

The majority of the title mol­ecule is disordered over two closely spaced locations; the conformation is approximately U-shaped. In the crystal, chains of mol­ecules extending along the *a*-axis direction are formed by C—H⋯O and C—H⋯Cl hydrogen bonds and are connected into a corrugated layer structure parallel to the *ab* plane by C—H⋯O hydrogen bonds and C—H⋯π(ring) inter­actions.

## Graphical abstract

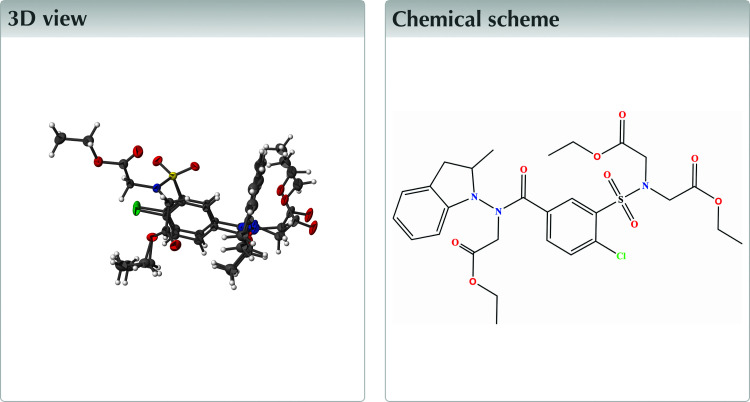

## Structure description

**Figure 1:**
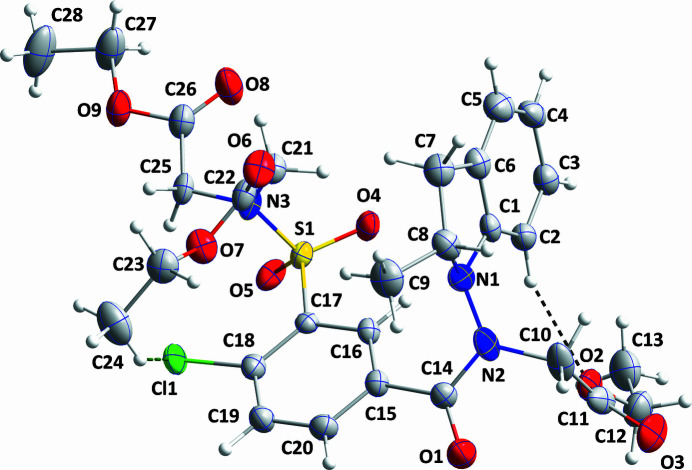
The title mol­ecule with labeling scheme and 50% probability ellipsoids. Only the major components of the disorder are shown. The intra­molecular C—H⋯O and C—H⋯Cl hydrogen bonds are depicted, respectively, by black and green dashed lines.

Indapamide, is a di­hydro-indole-based thia­zide-like diuretic used to manage heart failure and treat hypertension. Various activities are associated with indole derivatives, including anti­viral (Kadam & Wilson, 2016 ▸). As a continuation of our work in this area (*e.g*. Missioui *et al.*, 2022 ▸), the title compound was synthesized and its crystal structure is reported here (Fig. 1 ▸)

The mol­ecule adopts an approximate U-shaped conformation (Fig. 1 ▸) with the chloro­phenyl ring forming the base and the indolinyl and sulfamoyl groups the sides. The intra­molecular C24—H24*A*⋯Cl1 hydrogen bond (Table 1 ▸) may help to stabilize this conformation. A puckering analysis (Cremer & Pople, 1975 ▸) of the two components of the five-membered ring gave the parameters *Q*(2) = 0.247 (8) Å and φ(2) = 327 (2)° for the major component and *Q*(2) = 0.399 (9) Å and φ(2) = 329.6 (16)° for the other. The dihedral angle between the mean planes of the C1–C6 and C15–C20 rings is 86.2 (4)° while that between the two disordered components of the C1–C6 ring is 3.8 (5)° and that between the two disordered components of the C15—C20 ring is 5.68 (6)°.

**Figure 2:**
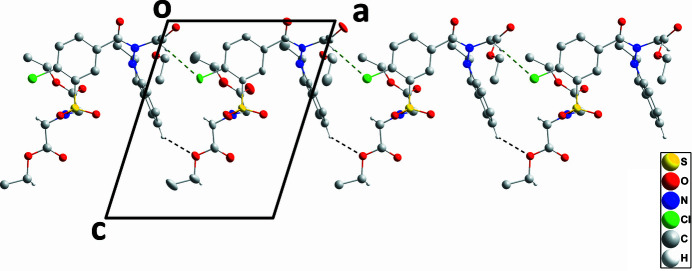
View of a portion of one chain seen along the *b*-axis direction with inter­molecular C—H⋯O and C—H⋯Cl hydrogen bonds depicted, respectively, by black and green dashed lines. Non-inter­acting hydrogen atoms are omitted.

**Figure 3:**
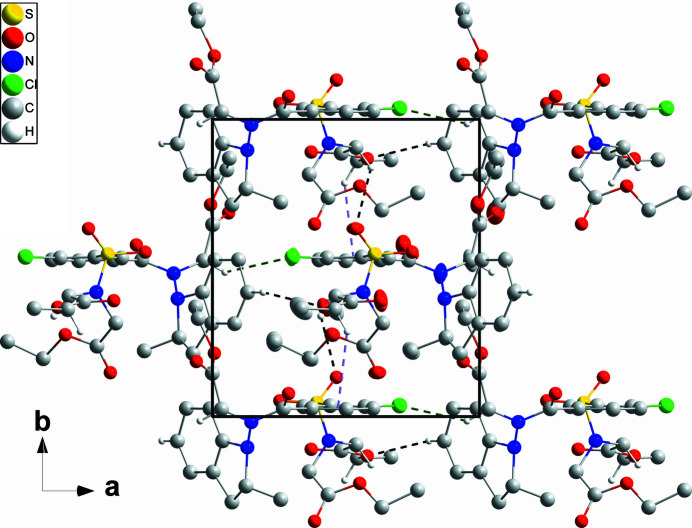
Packing viewed along the *c*-axis direction with hydrogen bonds depicted as in Fig. 2 ▸. C—H⋯π(ring) inter­actions are depicted by light-purple dashed lines and non-inter­acting hydrogen atoms are omitted.

In the crystal, C4—H4⋯O9 and C10—H10*A*⋯Cl1 hydrogen bonds (Table 1 ▸) cooperate to form chains of mol­ecules extending along the *a*-axis direction (Fig. 2 ▸). The chains are connected into corrugated layers parallel to the *ab* plane by C25—H25*A*⋯O5 hydrogen bonds and C27—H27*B*⋯*Cg*4 inter­actions (Table 1 ▸ and Fig. 3 ▸).

## Synthesis and crystallization

Indapamide (0.5 g, 1.36 mmol) and potassium bicarbonate (0.62 g, 4.5 mmol) were dissolved in di­methyl­formamide (10 mL), to which was added ethyl 2-bromo­acetate (0.69 g, 4.5 mmol). Under reflux, the reaction was stirred for 3 h at 80°C. When the starting reagents had reacted completely, distilled water (100 ml) was added. The product precipitated in solid form, was filtered, dried and recrystallized from ethanol solution to afford colorless blocks.

## Refinement

Crystal data, data collection and structure refinement details are summarized in Table 2 ▸. The ethyl 4-chloro­benzyl-*N*-(2-methyl­indolin-1-yl)glycinate portion of the mol­ecule is disordered over two partially resolved sets of sites in a 0.542 (3):0.458 (3) ratio. In addition, the C23—C24 ethyl group is disordered over two sets of sites in a 0.526 (12):0.474 (12) ratio. The two components of each disorder were refined with restraints that their geometries be comparable.

## Supplementary Material

Crystal structure: contains datablock(s) global, I. DOI: 10.1107/S2414314623006995/tk4094sup1.cif

Structure factors: contains datablock(s) I. DOI: 10.1107/S2414314623006995/tk4094Isup2.hkl

Supporting information file. DOI: 10.1107/S2414314623006995/tk4094Isup3.cml

CCDC reference: 2280340

Additional supporting information:  crystallographic information; 3D view; checkCIF report

## Figures and Tables

**Table 1:** Hydrogen-bond geometry (Å, °) *Cg*4 is the centroid of the major orientation of the C15–C20 benzene ring.

*D*—H⋯*A*	*D*—H	H⋯*A*	*D*⋯*A*	*D*—H⋯*A*
C2—H2⋯O2	0.95	2.37	3.198 (13)	145
C4—H4⋯O9^i^	0.95	2.48	3.262 (8)	139
C10—H10*A*⋯Cl1^i^	0.99	2.88	3.708 (11)	141
C24—H24*A*⋯Cl1	0.98	2.86	3.51 (2)	124
C25—H25*A*⋯O5^ii^	0.99	2.41	3.100 (5)	126
C27—H27*B*⋯*Cg*4^ii^	0.99	2.92	3.742 (7)	141

Symmetry codes: (i) 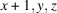 ; (ii) 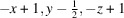 .

**Table 2:** Experimental details

Crystal data	
Chemical formula	C_28_H_34_ClN_3_O_9_S
*M*_r_	624.09
Crystal system, space group	Monoclinic, *P*2_1_
Temperature (K)	150
*a*, *b*, *c* (Å)	10.683 (2), 11.347 (2), 13.203 (3)
β (°)	107.491 (3)
*V* (Å^3^)	1526.4 (6)
*Z*	2
Radiation type	Mo *K*α
μ (mm^−1^)	0.25
Crystal size (mm)	0.36 × 0.26 × 0.06

Data collection	
Diffractometer	Bruker SMART APEX CCD Diffractometer
Absorption correction	Numerical (*SADABS*; Krause *et al.*, 2015 ▸)
*T*_min_, *T*_max_	0.92, 0.98
No. of measured, independent and observed [*I* > 2σ(*I*)] reflections	26691, 7798, 5986
*R*_int_	0.040
(sin θ/λ)_max_ (Å^−1^)	0.676

Refinement	
*R*[*F* ^2^ > 2σ(*F* ^2^)], *wR*(*F* ^2^), *S*	0.056, 0.154, 1.06
No. of reflections	7798
No. of parameters	448
No. of restraints	413
H-atom treatment	H-atom parameters constrained
Δρ_max_, Δρ_min_ (e Å^−3^)	0.67, −0.45
Absolute structure	Flack *x* determined using 2312 quotients [(*I* ^+^)−(*I* ^−^)]/[(*I* ^+^)+(*I* ^−^)] (Parsons *et al.*, 2013 ▸)
Absolute structure parameter	0.07 (3)

Computer programs: *APEX3* and *SAINT* (Bruker, 2020 ▸), *SHELXT2014/5* (Sheldrick, 2015*a* ▸), *SHELXL2018/3* (Sheldrick, 2015*b* ▸), *DIAMOND* (Brandenburg & Putz, 2012 ▸), and *SHELXTL* (Sheldrick, 2008 ▸).

## full crystallographic data

### Crystal data

**Table:**

C_28_H_34_ClN_3_O_9_S	*F*(000) = 656
*M**_r_* = 624.09	*D*_x_ = 1.358 Mg m^−^^3^
Monoclinic, *P*2_1_	Mo *K*α radiation, λ = 0.71073 Å
*a* = 10.683 (2) Å	Cell parameters from 8512 reflections
*b* = 11.347 (2) Å	θ = 2.4–26.5°
*c* = 13.203 (3) Å	µ = 0.25 mm^−^^1^
β = 107.491 (3)°	*T* = 150 K
*V* = 1526.4 (6) Å^3^	Plate, colourless
*Z* = 2	0.36 × 0.26 × 0.06 mm

### Data collection

**Table:**

Bruker SMART APEX CCD Diffractometer	5986 reflections with *I* > 2σ(*I*)
Radiation source: fine-focus sealed tube	*R*_int_ = 0.040
φ and ω scans	θ_max_ = 28.7°, θ_min_ = 1.6°
Absorption correction: numerical (*SADABS*; Krause *et al.*, 2015)	*h* = −14→14
*T*_min_ = 0.92, *T*_max_ = 0.98	*k* = −15→15
26691 measured reflections	*l* = −17→17
7798 independent reflections	

### Refinement

**Table:**

Refinement on *F*^2^	Secondary atom site location: difference Fourier map
Least-squares matrix: full	Hydrogen site location: inferred from neighbouring sites
*R*[*F*^2^ > 2σ(*F*^2^)] = 0.056	H-atom parameters constrained
*wR*(*F*^2^) = 0.154	*w* = 1/[σ^2^(*F*_o_^2^) + (0.0951*P*)^2^] where *P* = (*F*_o_^2^ + 2*F*_c_^2^)/3
*S* = 1.06	(Δ/σ)_max_ < 0.001
7798 reflections	Δρ_max_ = 0.67 e Å^−^^3^
448 parameters	Δρ_min_ = −0.45 e Å^−^^3^
413 restraints	Absolute structure: Flack *x* determined using 2312 quotients [(*I*^+^)-(*I*^-^)]/[(*I*^+^)+(*I*^-^)] (Parsons *et al.*, 2013)
Primary atom site location: dual	Absolute structure parameter: 0.07 (3)

### Special details

**Table:**

Experimental. The diffraction data were obtained from 3 sets of 400 frames, each of width 0.5 deg. in omega, collected at phi = 0.00, 90.00 and 180.00 deg. and 2 sets of 800 frames, each of width 0.45 deg in phi, collected at omega = -30.00 and 210.00 deg. The scan time was 25 sec/frame.
Geometry. All esds (except the esd in the dihedral angle between two l.s. planes) are estimated using the full covariance matrix. The cell esds are taken into account individually in the estimation of esds in distances, angles and torsion angles; correlations between esds in cell parameters are only used when they are defined by crystal symmetry. An approximate (isotropic) treatment of cell esds is used for estimating esds involving l.s. planes.
Refinement. Refinement of F^2^ against ALL reflections. The weighted R-factor wR and goodness of fit S are based on F^2^, conventional R-factors R are based on F, with F set to zero for negative F^2^. The threshold expression of F^2^ > 2sigma(F^2^) is used only for calculating R-factors(gt) etc. and is not relevant to the choice of reflections for refinement. R-factors based on F^2^ are statistically about twice as large as those based on F, and R- factors based on ALL data will be even larger. H-atoms attached to carbon were placed in calculated positions (C—H = 0.95 - 1.00 Å). All were included as riding contributions with isotropic displacement parameters 1.2 - 1.5 times those of the attached atoms. The ethyl 4-chlorobenzyl-N- (2-methylindolin-1-yl)glycinate portion of the molecule is disordered over two partially resolved sites in a 0.542 (3):0.458 (3) ratio. Also, the C23—C24 ethyl group is disordered over two sites in a 0.526 (12):0.474 (12) ratio. The two components of each disorder were refined with restraints that their geometries be comparable.

### Fractional atomic coordinates and isotropic or equivalent isotropic displacement parameters (Å^2^)

**Table:**

	*x*	*y*	*z*	*U*_iso_*/*U*_eq_	Occ. (<1)
S1	0.60808 (8)	0.54611 (11)	0.44326 (6)	0.0246 (2)	
O4	0.7476 (2)	0.5529 (3)	0.47429 (18)	0.0310 (5)	
O5	0.5355 (3)	0.6319 (2)	0.4804 (2)	0.0342 (7)	
O6	0.6174 (3)	0.1476 (3)	0.3450 (3)	0.0420 (7)	
O7	0.4485 (3)	0.2752 (3)	0.3126 (2)	0.0370 (7)	
O8	0.6303 (3)	0.3898 (4)	0.6935 (3)	0.0494 (8)	
O9	0.4203 (3)	0.3672 (3)	0.6856 (2)	0.0401 (7)	
N3	0.5647 (3)	0.4184 (3)	0.4747 (3)	0.0278 (7)	
Cl1	0.2994 (7)	0.5368 (15)	0.2927 (7)	0.0376 (19)	0.542 (3)
O1	0.7150 (6)	0.5773 (6)	0.0047 (4)	0.0422 (15)	0.542 (3)
O2	0.9930 (6)	0.7129 (5)	0.1760 (5)	0.0367 (12)	0.542 (3)
O3	1.0605 (7)	0.6851 (7)	0.0338 (5)	0.0565 (15)	0.542 (3)
N1	0.8695 (6)	0.3963 (4)	0.2179 (4)	0.0314 (9)	0.542 (3)
N2	0.8544 (5)	0.4813 (6)	0.1379 (5)	0.0395 (13)	0.542 (3)
C1	0.9622 (11)	0.4182 (5)	0.3174 (6)	0.0283 (10)	0.542 (3)
C2	1.0092 (19)	0.5264 (5)	0.3612 (9)	0.0316 (11)	0.542 (3)
H2	0.982257	0.597547	0.322925	0.038*	0.542 (3)
C3	1.0976 (8)	0.5272 (8)	0.4634 (6)	0.0342 (15)	0.542 (3)
H3	1.134539	0.598990	0.495918	0.041*	0.542 (3)
C4	1.1314 (7)	0.4175 (8)	0.5180 (6)	0.0369 (15)	0.542 (3)
H4	1.189407	0.417708	0.588456	0.044*	0.542 (3)
C5	1.0830 (9)	0.3126 (8)	0.4724 (7)	0.0425 (17)	0.542 (3)
H5	1.108653	0.240573	0.509459	0.051*	0.542 (3)
C6	0.9974 (8)	0.3133 (6)	0.3727 (6)	0.0314 (14)	0.542 (3)
C7	0.9270 (9)	0.2123 (6)	0.3051 (7)	0.0381 (14)	0.542 (3)
H7A	0.854404	0.182872	0.330333	0.046*	0.542 (3)
H7B	0.988117	0.146556	0.305549	0.046*	0.542 (3)
C8	0.8748 (7)	0.2658 (6)	0.1947 (6)	0.0344 (14)	0.542 (3)
H8	0.941488	0.253357	0.156400	0.041*	0.542 (3)
C9	0.7445 (9)	0.2209 (8)	0.1255 (7)	0.0489 (17)	0.542 (3)
H9A	0.718893	0.264456	0.058359	0.073*	0.542 (3)
H9B	0.677899	0.232090	0.162047	0.073*	0.542 (3)
H9C	0.751849	0.136822	0.111242	0.073*	0.542 (3)
C10	0.9664 (7)	0.5244 (8)	0.0989 (6)	0.0462 (19)	0.542 (3)
H10A	1.046523	0.485052	0.144029	0.055*	0.542 (3)
H10B	0.950032	0.491760	0.026578	0.055*	0.542 (3)
C11	1.0005 (8)	0.6456 (7)	0.0928 (6)	0.0353 (15)	0.542 (3)
C12	1.0536 (11)	0.8298 (9)	0.1908 (8)	0.0491 (19)	0.542 (3)
H12A	0.999331	0.885773	0.138173	0.059*	0.542 (3)
H12B	1.141981	0.826142	0.181313	0.059*	0.542 (3)
C13	1.0631 (11)	0.8686 (9)	0.2997 (9)	0.056 (2)	0.542 (3)
H13A	1.118669	0.938905	0.317384	0.084*	0.542 (3)
H13B	1.101680	0.805386	0.350035	0.084*	0.542 (3)
H13C	0.975245	0.887158	0.304028	0.084*	0.542 (3)
C14	0.7348 (5)	0.5262 (7)	0.0897 (4)	0.0273 (13)	0.542 (3)
C15	0.6294 (4)	0.5271 (5)	0.1450 (3)	0.0241 (12)	0.542 (3)
C16	0.6595 (4)	0.5333 (6)	0.2548 (3)	0.0229 (12)	0.542 (3)
H16	0.748523	0.535298	0.297764	0.028*	0.542 (3)
C17	0.5593 (5)	0.5365 (6)	0.30187 (19)	0.0211 (11)	0.542 (3)
C18	0.4290 (5)	0.5335 (6)	0.2390 (3)	0.0276 (13)	0.542 (3)
C19	0.3989 (4)	0.5273 (6)	0.1292 (3)	0.0313 (14)	0.542 (3)
H19	0.309902	0.525314	0.086237	0.038*	0.542 (3)
C20	0.4991 (4)	0.5242 (5)	0.08213 (19)	0.0301 (13)	0.542 (3)
H20	0.478550	0.519962	0.007036	0.036*	0.542 (3)
Cl1A	0.2959 (9)	0.5595 (18)	0.2923 (8)	0.0376 (19)	0.458 (3)
O1A	0.7240 (7)	0.6425 (7)	0.0167 (5)	0.0422 (15)	0.458 (3)
O2A	0.9978 (7)	0.6768 (6)	0.1420 (6)	0.0367 (12)	0.458 (3)
O3A	1.0740 (8)	0.6132 (8)	0.0093 (6)	0.0565 (15)	0.458 (3)
N1A	0.8383 (6)	0.4156 (5)	0.2036 (4)	0.0314 (9)	0.458 (3)
N2A	0.8422 (6)	0.5135 (6)	0.1379 (5)	0.0395 (13)	0.458 (3)
C1A	0.9428 (13)	0.4089 (5)	0.2993 (7)	0.0283 (10)	0.458 (3)
C2A	1.008 (2)	0.5001 (6)	0.3632 (11)	0.0316 (11)	0.458 (3)
H2A	0.992996	0.579694	0.340541	0.038*	0.458 (3)
C3A	1.0957 (10)	0.4718 (9)	0.4620 (7)	0.0342 (15)	0.458 (3)
H3A	1.140601	0.531675	0.509339	0.041*	0.458 (3)
C4A	1.1160 (10)	0.3498 (9)	0.4900 (7)	0.0369 (15)	0.458 (3)
H4A	1.177243	0.329063	0.556131	0.044*	0.458 (3)
C5A	1.0501 (10)	0.2627 (8)	0.4244 (8)	0.0425 (17)	0.458 (3)
H5A	1.062510	0.182548	0.445958	0.051*	0.458 (3)
C6A	0.9667 (9)	0.2915 (6)	0.3282 (7)	0.0314 (14)	0.458 (3)
C7A	0.8798 (9)	0.2147 (6)	0.2421 (8)	0.0381 (14)	0.458 (3)
H7C	0.803112	0.185759	0.262288	0.046*	0.458 (3)
H7D	0.928945	0.146341	0.226989	0.046*	0.458 (3)
C8A	0.8374 (8)	0.2972 (7)	0.1472 (7)	0.0344 (14)	0.458 (3)
H8A	0.905918	0.297988	0.109635	0.041*	0.458 (3)
C9A	0.7047 (9)	0.2781 (9)	0.0680 (8)	0.0489 (17)	0.458 (3)
H9D	0.687923	0.339864	0.013628	0.073*	0.458 (3)
H9E	0.637145	0.281223	0.104319	0.073*	0.458 (3)
H9F	0.702486	0.200856	0.034308	0.073*	0.458 (3)
C10A	0.9400 (9)	0.4912 (8)	0.0750 (7)	0.0462 (19)	0.458 (3)
H10C	0.997541	0.424066	0.106883	0.055*	0.458 (3)
H10D	0.891210	0.470055	0.000840	0.055*	0.458 (3)
C11A	1.0188 (8)	0.5934 (9)	0.0752 (7)	0.0353 (15)	0.458 (3)
C12A	1.0608 (11)	0.7912 (9)	0.1428 (10)	0.0491 (19)	0.458 (3)
H12C	1.021000	0.833033	0.074944	0.059*	0.458 (3)
H12D	1.155707	0.780818	0.152451	0.059*	0.458 (3)
C13A	1.0410 (13)	0.8592 (10)	0.2321 (12)	0.056 (2)	0.458 (3)
H13D	1.073716	0.939632	0.230606	0.084*	0.458 (3)
H13E	1.088938	0.821364	0.299294	0.084*	0.458 (3)
H13F	0.947231	0.861575	0.225608	0.084*	0.458 (3)
C14A	0.7347 (6)	0.5776 (9)	0.0926 (5)	0.0273 (13)	0.458 (3)
C15A	0.6263 (5)	0.5714 (6)	0.1452 (3)	0.0241 (12)	0.458 (3)
C16A	0.6562 (5)	0.5654 (7)	0.2550 (3)	0.0229 (12)	0.458 (3)
H16A	0.745202	0.563497	0.298079	0.028*	0.458 (3)
C17A	0.5559 (6)	0.5623 (8)	0.3020 (2)	0.0211 (11)	0.458 (3)
C18A	0.4257 (6)	0.5651 (8)	0.2390 (4)	0.0276 (13)	0.458 (3)
C19A	0.3958 (4)	0.5711 (7)	0.1291 (3)	0.0313 (14)	0.458 (3)
H19A	0.306771	0.573059	0.086099	0.038*	0.458 (3)
C20A	0.4961 (5)	0.5743 (7)	0.0822 (2)	0.0301 (13)	0.458 (3)
H20A	0.475641	0.578374	0.007135	0.036*	0.458 (3)
C21	0.6376 (4)	0.3145 (3)	0.4586 (3)	0.0301 (8)	
H21A	0.659298	0.266133	0.524055	0.036*	
H21B	0.721346	0.340976	0.448656	0.036*	
C22	0.5672 (4)	0.2368 (3)	0.3654 (3)	0.0324 (9)	
C23	0.3658 (10)	0.1945 (11)	0.2343 (10)	0.040 (2)	0.526 (12)
H23A	0.355884	0.119007	0.268714	0.048*	0.526 (12)
H23B	0.406677	0.177976	0.177690	0.048*	0.526 (12)
C24	0.2319 (10)	0.2520 (12)	0.1868 (11)	0.058 (2)	0.526 (12)
H24A	0.243520	0.331157	0.161230	0.088*	0.526 (12)
H24B	0.186810	0.257710	0.241208	0.088*	0.526 (12)
H24C	0.179278	0.204217	0.127370	0.088*	0.526 (12)
C23A	0.3757 (11)	0.2112 (15)	0.2169 (10)	0.040 (2)	0.474 (12)
H23C	0.417236	0.134334	0.211744	0.048*	0.474 (12)
H23D	0.368740	0.257769	0.152071	0.048*	0.474 (12)
C24A	0.2418 (12)	0.1949 (14)	0.2341 (12)	0.058 (2)	0.474 (12)
H24D	0.206690	0.271934	0.245321	0.088*	0.474 (12)
H24E	0.251091	0.145121	0.296619	0.088*	0.474 (12)
H24F	0.181707	0.157156	0.171493	0.088*	0.474 (12)
C25	0.4624 (4)	0.4050 (3)	0.5260 (3)	0.0283 (8)	
H25A	0.405925	0.337290	0.493872	0.034*	
H25B	0.406843	0.476623	0.512948	0.034*	
C26	0.5177 (4)	0.3856 (4)	0.6440 (3)	0.0337 (9)	
C27	0.4571 (5)	0.3598 (6)	0.8008 (4)	0.0537 (13)	
H27A	0.514494	0.426712	0.833385	0.064*	
H27B	0.504902	0.285421	0.825727	0.064*	
C28	0.3319 (6)	0.3635 (6)	0.8307 (5)	0.0665 (18)	
H28A	0.288818	0.439891	0.810204	0.100*	
H28B	0.351927	0.352855	0.907560	0.100*	
H28C	0.273308	0.300324	0.793714	0.100*	

### Atomic displacement parameters (Å^2^)

**Table:**

	*U*^11^	*U*^22^	*U*^33^	*U*^12^	*U*^13^	*U*^23^
S1	0.0278 (4)	0.0230 (4)	0.0258 (4)	−0.0039 (4)	0.0123 (3)	−0.0033 (4)
O4	0.0294 (13)	0.0345 (13)	0.0293 (11)	−0.0110 (13)	0.0093 (10)	−0.0042 (12)
O5	0.0490 (18)	0.0239 (13)	0.0383 (15)	0.0003 (12)	0.0263 (14)	−0.0012 (11)
O6	0.0490 (18)	0.0275 (15)	0.057 (2)	0.0010 (13)	0.0278 (15)	−0.0112 (13)
O7	0.0382 (17)	0.0319 (15)	0.0413 (16)	−0.0013 (12)	0.0123 (13)	−0.0134 (12)
O8	0.0308 (18)	0.079 (3)	0.0393 (17)	−0.0068 (16)	0.0117 (14)	−0.0076 (16)
O9	0.0353 (17)	0.0540 (19)	0.0361 (16)	−0.0125 (14)	0.0186 (13)	−0.0095 (14)
N3	0.0320 (18)	0.0217 (15)	0.0363 (17)	−0.0046 (12)	0.0202 (14)	−0.0060 (13)
Cl1	0.0236 (5)	0.053 (6)	0.0403 (5)	0.0055 (13)	0.0156 (4)	0.0047 (14)
O1	0.037 (2)	0.062 (4)	0.029 (2)	0.000 (3)	0.0123 (17)	0.004 (2)
O2	0.0313 (18)	0.052 (3)	0.034 (3)	0.001 (2)	0.020 (2)	0.000 (2)
O3	0.052 (3)	0.087 (4)	0.047 (3)	0.003 (3)	0.039 (2)	0.005 (3)
N1	0.0309 (10)	0.0318 (10)	0.0317 (10)	0.0006 (4)	0.0097 (5)	−0.0010 (4)
N2	0.025 (2)	0.068 (3)	0.0295 (17)	0.0047 (19)	0.0131 (15)	−0.008 (2)
C1	0.018 (3)	0.035 (2)	0.033 (2)	−0.0011 (16)	0.0099 (19)	−0.0027 (17)
C2	0.0267 (19)	0.036 (3)	0.0322 (18)	0.000 (3)	0.0094 (16)	0.002 (2)
C3	0.028 (2)	0.040 (4)	0.036 (2)	−0.001 (3)	0.0118 (18)	−0.005 (3)
C4	0.027 (3)	0.050 (3)	0.033 (3)	0.007 (3)	0.007 (2)	0.004 (2)
C5	0.038 (3)	0.043 (3)	0.048 (3)	0.009 (3)	0.014 (3)	0.004 (2)
C6	0.029 (3)	0.033 (3)	0.035 (3)	0.002 (2)	0.013 (3)	−0.007 (2)
C7	0.039 (3)	0.033 (2)	0.044 (3)	0.001 (2)	0.015 (3)	−0.007 (3)
C8	0.031 (3)	0.040 (3)	0.032 (3)	0.005 (2)	0.010 (2)	−0.007 (2)
C9	0.048 (4)	0.049 (4)	0.048 (4)	−0.010 (3)	0.012 (3)	−0.016 (3)
C10	0.032 (3)	0.080 (4)	0.028 (3)	−0.015 (3)	0.010 (2)	−0.020 (3)
C11	0.031 (3)	0.049 (3)	0.032 (3)	0.016 (2)	0.018 (2)	0.005 (3)
C12	0.048 (3)	0.058 (3)	0.048 (4)	−0.007 (3)	0.024 (3)	0.002 (3)
C13	0.044 (4)	0.056 (4)	0.066 (5)	−0.014 (3)	0.014 (4)	−0.010 (4)
C14	0.0283 (19)	0.030 (3)	0.0235 (17)	0.001 (3)	0.0083 (15)	−0.004 (3)
C15	0.0245 (18)	0.022 (3)	0.0281 (16)	0.001 (2)	0.0110 (14)	−0.004 (2)
C16	0.0214 (16)	0.021 (3)	0.0282 (15)	0.002 (2)	0.0106 (13)	−0.003 (2)
C17	0.0230 (16)	0.017 (3)	0.0243 (14)	0.0040 (19)	0.0084 (12)	0.0019 (17)
C18	0.0267 (17)	0.025 (3)	0.0336 (16)	0.004 (2)	0.0128 (14)	0.002 (2)
C19	0.0247 (18)	0.035 (4)	0.0331 (18)	0.002 (3)	0.0071 (15)	0.000 (3)
C20	0.032 (2)	0.032 (3)	0.0252 (17)	0.001 (3)	0.0074 (16)	−0.002 (3)
Cl1A	0.0236 (5)	0.053 (6)	0.0403 (5)	0.0055 (13)	0.0156 (4)	0.0047 (14)
O1A	0.037 (2)	0.062 (4)	0.029 (2)	0.000 (3)	0.0123 (17)	0.004 (2)
O2A	0.0313 (18)	0.052 (3)	0.034 (3)	0.001 (2)	0.020 (2)	0.000 (2)
O3A	0.052 (3)	0.087 (4)	0.047 (3)	0.003 (3)	0.039 (2)	0.005 (3)
N1A	0.0309 (10)	0.0318 (10)	0.0317 (10)	0.0006 (4)	0.0097 (5)	−0.0010 (4)
N2A	0.025 (2)	0.068 (3)	0.0295 (17)	0.0047 (19)	0.0131 (15)	−0.008 (2)
C1A	0.018 (3)	0.035 (2)	0.033 (2)	−0.0011 (16)	0.0099 (19)	−0.0027 (17)
C2A	0.0267 (19)	0.036 (3)	0.0322 (18)	0.000 (3)	0.0094 (16)	0.002 (2)
C3A	0.028 (2)	0.040 (4)	0.036 (2)	−0.001 (3)	0.0118 (18)	−0.005 (3)
C4A	0.027 (3)	0.050 (3)	0.033 (3)	0.007 (3)	0.007 (2)	0.004 (2)
C5A	0.038 (3)	0.043 (3)	0.048 (3)	0.009 (3)	0.014 (3)	0.004 (2)
C6A	0.029 (3)	0.033 (3)	0.035 (3)	0.002 (2)	0.013 (3)	−0.007 (2)
C7A	0.039 (3)	0.033 (2)	0.044 (3)	0.001 (2)	0.015 (3)	−0.007 (3)
C8A	0.031 (3)	0.040 (3)	0.032 (3)	0.005 (2)	0.010 (2)	−0.007 (2)
C9A	0.048 (4)	0.049 (4)	0.048 (4)	−0.010 (3)	0.012 (3)	−0.016 (3)
C10A	0.032 (3)	0.080 (4)	0.028 (3)	−0.015 (3)	0.010 (2)	−0.020 (3)
C11A	0.031 (3)	0.049 (3)	0.032 (3)	0.016 (2)	0.018 (2)	0.005 (3)
C12A	0.048 (3)	0.058 (3)	0.048 (4)	−0.007 (3)	0.024 (3)	0.002 (3)
C13A	0.044 (4)	0.056 (4)	0.066 (5)	−0.014 (3)	0.014 (4)	−0.010 (4)
C14A	0.0283 (19)	0.030 (3)	0.0235 (17)	0.001 (3)	0.0083 (15)	−0.004 (3)
C15A	0.0245 (18)	0.022 (3)	0.0281 (16)	0.001 (2)	0.0110 (14)	−0.004 (2)
C16A	0.0214 (16)	0.021 (3)	0.0282 (15)	0.002 (2)	0.0106 (13)	−0.003 (2)
C17A	0.0230 (16)	0.017 (3)	0.0243 (14)	0.0040 (19)	0.0084 (12)	0.0019 (17)
C18A	0.0267 (17)	0.025 (3)	0.0336 (16)	0.004 (2)	0.0128 (14)	0.002 (2)
C19A	0.0247 (18)	0.035 (4)	0.0331 (18)	0.002 (3)	0.0071 (15)	0.000 (3)
C20A	0.032 (2)	0.032 (3)	0.0252 (17)	0.001 (3)	0.0074 (16)	−0.002 (3)
C21	0.031 (2)	0.0236 (17)	0.038 (2)	−0.0001 (14)	0.0149 (16)	−0.0050 (14)
C22	0.035 (2)	0.0268 (18)	0.043 (2)	−0.0036 (15)	0.0227 (18)	−0.0041 (15)
C23	0.044 (3)	0.039 (3)	0.038 (3)	−0.005 (2)	0.013 (2)	−0.010 (3)
C24	0.041 (3)	0.064 (5)	0.065 (5)	−0.004 (4)	0.008 (4)	−0.025 (4)
C23A	0.044 (3)	0.039 (3)	0.038 (3)	−0.005 (2)	0.013 (2)	−0.010 (3)
C24A	0.041 (3)	0.064 (5)	0.065 (5)	−0.004 (4)	0.008 (4)	−0.025 (4)
C25	0.027 (2)	0.0231 (18)	0.040 (2)	−0.0057 (14)	0.0179 (16)	−0.0044 (14)
C26	0.033 (2)	0.033 (2)	0.040 (2)	−0.0060 (17)	0.0172 (18)	−0.0073 (16)
C27	0.055 (3)	0.075 (4)	0.038 (2)	−0.025 (3)	0.025 (2)	−0.018 (2)
C28	0.079 (4)	0.083 (4)	0.057 (3)	−0.031 (3)	0.049 (3)	−0.029 (3)

### Geometric parameters (Å, º)

**Table:**

S1—O5	1.421 (3)	N1A—C1A	1.414 (7)
S1—O4	1.424 (3)	N1A—N2A	1.418 (7)
S1—N3	1.613 (3)	N1A—C8A	1.535 (7)
S1—C17	1.784 (3)	N2A—C14A	1.339 (7)
S1—C17A	1.789 (3)	N2A—C10A	1.538 (7)
O6—C22	1.213 (5)	C1A—C2A	1.382 (7)
O7—C22	1.324 (5)	C1A—C6A	1.388 (7)
O7—C23A	1.462 (6)	C2A—C3A	1.397 (7)
O7—C23	1.462 (6)	C2A—H2A	0.9500
O8—C26	1.184 (5)	C3A—C4A	1.432 (12)
O9—C26	1.331 (5)	C3A—H3A	0.9500
O9—C27	1.454 (6)	C4A—C5A	1.363 (12)
N3—C25	1.457 (5)	C4A—H4A	0.9500
N3—C21	1.463 (5)	C5A—C6A	1.355 (11)
Cl1—C18	1.735 (3)	C5A—H5A	0.9500
O1—C14	1.223 (7)	C6A—C7A	1.510 (10)
O2—C11	1.361 (9)	C7A—C8A	1.520 (11)
O2—C12	1.463 (10)	C7A—H7C	0.9900
O3—C11	1.233 (7)	C7A—H7D	0.9900
N1—N2	1.403 (6)	C8A—C9A	1.503 (10)
N1—C1	1.410 (6)	C8A—H8A	1.0000
N1—C8	1.517 (7)	C9A—H9D	0.9800
N2—C14	1.344 (7)	C9A—H9E	0.9800
N2—C10	1.518 (7)	C9A—H9F	0.9800
C1—C2	1.384 (7)	C10A—C11A	1.433 (11)
C1—C6	1.388 (7)	C10A—H10C	0.9900
C2—C3	1.395 (7)	C10A—H10D	0.9900
C2—H2	0.9500	C12A—C13A	1.475 (12)
C3—C4	1.429 (11)	C12A—H12C	0.9900
C3—H3	0.9500	C12A—H12D	0.9900
C4—C5	1.364 (12)	C13A—H13D	0.9800
C4—H4	0.9500	C13A—H13E	0.9800
C5—C6	1.358 (11)	C13A—H13F	0.9800
C5—H5	0.9500	C14A—C15A	1.520 (5)
C6—C7	1.508 (10)	C15A—C16A	1.3900
C7—C8	1.522 (11)	C15A—C20A	1.3900
C7—H7A	0.9900	C16A—C17A	1.3900
C7—H7B	0.9900	C16A—H16A	0.9500
C8—C9	1.505 (10)	C17A—C18A	1.3900
C8—H8	1.0000	C18A—C19A	1.3900
C9—H9A	0.9800	C19A—C20A	1.3900
C9—H9B	0.9800	C19A—H19A	0.9500
C9—H9C	0.9800	C20A—H20A	0.9500
C10—C11	1.431 (11)	C21—C22	1.517 (5)
C10—H10A	0.9900	C21—H21A	0.9900
C10—H10B	0.9900	C21—H21B	0.9900
C12—C13	1.478 (12)	C23—C24	1.526 (11)
C12—H12A	0.9900	C23—H23A	0.9900
C12—H12B	0.9900	C23—H23B	0.9900
C13—H13A	0.9800	C24—H24A	0.9800
C13—H13B	0.9800	C24—H24B	0.9800
C13—H13C	0.9800	C24—H24C	0.9800
C14—C15	1.515 (5)	C23A—C24A	1.525 (11)
C15—C16	1.3900	C23A—H23C	0.9900
C15—C20	1.3900	C23A—H23D	0.9900
C16—C17	1.3900	C24A—H24D	0.9800
C16—H16	0.9500	C24A—H24E	0.9800
C17—C18	1.3900	C24A—H24F	0.9800
C18—C19	1.3900	C25—C26	1.508 (6)
C19—C20	1.3900	C25—H25A	0.9900
C19—H19	0.9500	C25—H25B	0.9900
C20—H20	0.9500	C27—C28	1.505 (7)
Cl1A—C18A	1.735 (3)	C27—H27A	0.9900
O1A—C14A	1.221 (7)	C27—H27B	0.9900
O2A—C11A	1.357 (9)	C28—H28A	0.9800
O2A—C12A	1.461 (10)	C28—H28B	0.9800
O3A—C11A	1.211 (7)	C28—H28C	0.9800
			
O5—S1—O4	119.78 (18)	C6A—C5A—H5A	120.4
O5—S1—N3	107.34 (17)	C4A—C5A—H5A	120.4
O4—S1—N3	109.32 (18)	C5A—C6A—C1A	120.3 (7)
O5—S1—C17	112.6 (2)	C5A—C6A—C7A	130.4 (7)
O4—S1—C17	104.8 (2)	C1A—C6A—C7A	109.1 (6)
N3—S1—C17	101.4 (3)	C6A—C7A—C8A	103.2 (5)
O5—S1—C17A	105.0 (3)	C6A—C7A—H7C	111.1
O4—S1—C17A	105.4 (2)	C8A—C7A—H7C	111.1
N3—S1—C17A	109.7 (3)	C6A—C7A—H7D	111.1
C22—O7—C23A	117.9 (7)	C8A—C7A—H7D	111.1
C22—O7—C23	116.5 (6)	H7C—C7A—H7D	109.1
C26—O9—C27	116.2 (3)	C9A—C8A—C7A	118.0 (7)
C25—N3—C21	119.3 (3)	C9A—C8A—N1A	109.3 (6)
C25—N3—S1	121.8 (3)	C7A—C8A—N1A	100.5 (5)
C21—N3—S1	118.7 (2)	C9A—C8A—H8A	109.5
C11—O2—C12	119.0 (6)	C7A—C8A—H8A	109.5
N2—N1—C1	118.0 (4)	N1A—C8A—H8A	109.5
N2—N1—C8	121.5 (5)	C8A—C9A—H9D	109.5
C1—N1—C8	107.6 (5)	C8A—C9A—H9E	109.5
C14—N2—N1	119.6 (5)	H9D—C9A—H9E	109.5
C14—N2—C10	116.8 (5)	C8A—C9A—H9F	109.5
N1—N2—C10	123.5 (5)	H9D—C9A—H9F	109.5
C2—C1—C6	122.3 (5)	H9E—C9A—H9F	109.5
C2—C1—N1	127.6 (4)	C11A—C10A—N2A	111.1 (6)
C6—C1—N1	110.0 (5)	C11A—C10A—H10C	109.4
C1—C2—C3	117.6 (6)	N2A—C10A—H10C	109.4
C1—C2—H2	121.2	C11A—C10A—H10D	109.4
C3—C2—H2	121.2	N2A—C10A—H10D	109.4
C2—C3—C4	118.5 (7)	H10C—C10A—H10D	108.0
C2—C3—H3	120.7	O3A—C11A—O2A	123.3 (9)
C4—C3—H3	120.7	O3A—C11A—C10A	124.2 (9)
C5—C4—C3	122.1 (6)	O2A—C11A—C10A	110.7 (5)
C5—C4—H4	118.9	O2A—C12A—C13A	107.1 (7)
C3—C4—H4	118.9	O2A—C12A—H12C	110.3
C6—C5—C4	118.6 (7)	C13A—C12A—H12C	110.3
C6—C5—H5	120.7	O2A—C12A—H12D	110.3
C4—C5—H5	120.7	C13A—C12A—H12D	110.3
C5—C6—C1	120.8 (7)	H12C—C12A—H12D	108.6
C5—C6—C7	129.8 (6)	C12A—C13A—H13D	109.5
C1—C6—C7	109.4 (6)	C12A—C13A—H13E	109.5
C6—C7—C8	103.8 (5)	H13D—C13A—H13E	109.5
C6—C7—H7A	111.0	C12A—C13A—H13F	109.5
C8—C7—H7A	111.0	H13D—C13A—H13F	109.5
C6—C7—H7B	111.0	H13E—C13A—H13F	109.5
C8—C7—H7B	111.0	O1A—C14A—N2A	123.3 (5)
H7A—C7—H7B	109.0	O1A—C14A—C15A	120.4 (6)
C9—C8—N1	111.7 (6)	N2A—C14A—C15A	116.1 (5)
C9—C8—C7	116.8 (7)	C16A—C15A—C20A	120.0
N1—C8—C7	102.8 (5)	C16A—C15A—C14A	120.7 (3)
C9—C8—H8	108.4	C20A—C15A—C14A	119.2 (3)
N1—C8—H8	108.4	C15A—C16A—C17A	120.0
C7—C8—H8	108.4	C15A—C16A—H16A	120.0
C8—C9—H9A	109.5	C17A—C16A—H16A	120.0
C8—C9—H9B	109.5	C16A—C17A—C18A	120.0
H9A—C9—H9B	109.5	C16A—C17A—S1	115.3 (3)
C8—C9—H9C	109.5	C18A—C17A—S1	124.6 (3)
H9A—C9—H9C	109.5	C19A—C18A—C17A	120.0
H9B—C9—H9C	109.5	C19A—C18A—Cl1A	117.7 (3)
C11—C10—N2	124.7 (7)	C17A—C18A—Cl1A	122.3 (3)
C11—C10—H10A	106.2	C18A—C19A—C20A	120.0
N2—C10—H10A	106.2	C18A—C19A—H19A	120.0
C11—C10—H10B	106.2	C20A—C19A—H19A	120.0
N2—C10—H10B	106.2	C19A—C20A—C15A	120.0
H10A—C10—H10B	106.4	C19A—C20A—H20A	120.0
O3—C11—O2	118.3 (7)	C15A—C20A—H20A	120.0
O3—C11—C10	125.2 (8)	N3—C21—C22	115.5 (3)
O2—C11—C10	114.5 (5)	N3—C21—H21A	108.4
O2—C12—C13	107.3 (7)	C22—C21—H21A	108.4
O2—C12—H12A	110.3	N3—C21—H21B	108.4
C13—C12—H12A	110.3	C22—C21—H21B	108.4
O2—C12—H12B	110.3	H21A—C21—H21B	107.5
C13—C12—H12B	110.3	O6—C22—O7	125.1 (4)
H12A—C12—H12B	108.5	O6—C22—C21	121.4 (4)
C12—C13—H13A	109.5	O7—C22—C21	113.5 (3)
C12—C13—H13B	109.5	O7—C23—C24	108.4 (8)
H13A—C13—H13B	109.5	O7—C23—H23A	110.0
C12—C13—H13C	109.5	C24—C23—H23A	110.0
H13A—C13—H13C	109.5	O7—C23—H23B	110.0
H13B—C13—H13C	109.5	C24—C23—H23B	110.0
O1—C14—N2	119.5 (5)	H23A—C23—H23B	108.4
O1—C14—C15	119.3 (5)	C23—C24—H24A	109.5
N2—C14—C15	120.8 (5)	C23—C24—H24B	109.5
C16—C15—C20	120.0	H24A—C24—H24B	109.5
C16—C15—C14	122.1 (3)	C23—C24—H24C	109.5
C20—C15—C14	117.9 (3)	H24A—C24—H24C	109.5
C17—C16—C15	120.0	H24B—C24—H24C	109.5
C17—C16—H16	120.0	O7—C23A—C24A	102.2 (8)
C15—C16—H16	120.0	O7—C23A—H23C	111.3
C16—C17—C18	120.0	C24A—C23A—H23C	111.3
C16—C17—S1	116.6 (3)	O7—C23A—H23D	111.3
C18—C17—S1	123.4 (3)	C24A—C23A—H23D	111.3
C17—C18—C19	120.0	H23C—C23A—H23D	109.2
C17—C18—Cl1	122.3 (2)	C23A—C24A—H24D	109.5
C19—C18—Cl1	117.7 (2)	C23A—C24A—H24E	109.5
C20—C19—C18	120.0	H24D—C24A—H24E	109.5
C20—C19—H19	120.0	C23A—C24A—H24F	109.5
C18—C19—H19	120.0	H24D—C24A—H24F	109.5
C19—C20—C15	120.0	H24E—C24A—H24F	109.5
C19—C20—H20	120.0	N3—C25—C26	112.3 (3)
C15—C20—H20	120.0	N3—C25—H25A	109.1
C11A—O2A—C12A	117.3 (7)	C26—C25—H25A	109.1
C1A—N1A—N2A	115.3 (5)	N3—C25—H25B	109.1
C1A—N1A—C8A	105.9 (5)	C26—C25—H25B	109.1
N2A—N1A—C8A	112.7 (5)	H25A—C25—H25B	107.9
C14A—N2A—N1A	121.3 (5)	O8—C26—O9	124.6 (4)
C14A—N2A—C10A	118.6 (5)	O8—C26—C25	125.6 (4)
N1A—N2A—C10A	110.5 (5)	O9—C26—C25	109.7 (3)
C2A—C1A—C6A	122.2 (6)	O9—C27—C28	106.9 (4)
C2A—C1A—N1A	128.4 (5)	O9—C27—H27A	110.3
C6A—C1A—N1A	109.1 (5)	C28—C27—H27A	110.3
C1A—C2A—C3A	118.1 (6)	O9—C27—H27B	110.3
C1A—C2A—H2A	120.9	C28—C27—H27B	110.3
C3A—C2A—H2A	120.9	H27A—C27—H27B	108.6
C2A—C3A—C4A	118.1 (7)	C27—C28—H28A	109.5
C2A—C3A—H3A	121.0	C27—C28—H28B	109.5
C4A—C3A—H3A	121.0	H28A—C28—H28B	109.5
C5A—C4A—C3A	121.9 (7)	C27—C28—H28C	109.5
C5A—C4A—H4A	119.1	H28A—C28—H28C	109.5
C3A—C4A—H4A	119.1	H28B—C28—H28C	109.5
C6A—C5A—C4A	119.3 (7)		
			
O5—S1—N3—C25	3.4 (3)	C8A—N1A—C1A—C2A	160 (2)
O4—S1—N3—C25	134.7 (3)	N2A—N1A—C1A—C6A	−150.5 (9)
C17—S1—N3—C25	−114.9 (3)	C8A—N1A—C1A—C6A	−25.1 (12)
C17A—S1—N3—C25	−110.1 (4)	C6A—C1A—C2A—C3A	−3 (3)
O5—S1—N3—C21	−171.8 (3)	N1A—C1A—C2A—C3A	171.0 (16)
O4—S1—N3—C21	−40.4 (3)	C1A—C2A—C3A—C4A	2 (3)
C17—S1—N3—C21	69.9 (4)	C2A—C3A—C4A—C5A	−2 (2)
C17A—S1—N3—C21	74.7 (4)	C3A—C4A—C5A—C6A	2.7 (18)
C1—N1—N2—C14	−125.3 (8)	C4A—C5A—C6A—C1A	−3.8 (17)
C8—N1—N2—C14	98.0 (8)	C4A—C5A—C6A—C7A	−178.2 (10)
C1—N1—N2—C10	56.6 (11)	C2A—C1A—C6A—C5A	4 (2)
C8—N1—N2—C10	−80.0 (9)	N1A—C1A—C6A—C5A	−171.0 (10)
N2—N1—C1—C2	24 (2)	C2A—C1A—C6A—C7A	179.6 (19)
C8—N1—C1—C2	166.5 (17)	N1A—C1A—C6A—C7A	4.6 (14)
N2—N1—C1—C6	−159.4 (8)	C5A—C6A—C7A—C8A	−167.4 (10)
C8—N1—C1—C6	−17.3 (11)	C1A—C6A—C7A—C8A	17.7 (12)
C6—C1—C2—C3	2 (3)	C6A—C7A—C8A—C9A	−149.1 (7)
N1—C1—C2—C3	177.6 (14)	C6A—C7A—C8A—N1A	−30.5 (8)
C1—C2—C3—C4	−2 (3)	C1A—N1A—C8A—C9A	159.4 (9)
C2—C3—C4—C5	1.9 (18)	N2A—N1A—C8A—C9A	−73.6 (8)
C3—C4—C5—C6	−1.7 (14)	C1A—N1A—C8A—C7A	34.5 (8)
C4—C5—C6—C1	1.5 (15)	N2A—N1A—C8A—C7A	161.6 (6)
C4—C5—C6—C7	−178.3 (8)	C14A—N2A—C10A—C11A	76.9 (10)
C2—C1—C6—C5	−2 (2)	N1A—N2A—C10A—C11A	−136.3 (7)
N1—C1—C6—C5	−178.1 (9)	C12A—O2A—C11A—O3A	−8.4 (14)
C2—C1—C6—C7	178.3 (16)	C12A—O2A—C11A—C10A	−173.8 (9)
N1—C1—C6—C7	1.8 (12)	N2A—C10A—C11A—O3A	−157.4 (9)
C5—C6—C7—C8	−166.1 (9)	N2A—C10A—C11A—O2A	7.9 (11)
C1—C6—C7—C8	14.1 (10)	C11A—O2A—C12A—C13A	−171.3 (10)
N2—N1—C8—C9	−68.4 (8)	N1A—N2A—C14A—O1A	−161.4 (9)
C1—N1—C8—C9	151.1 (8)	C10A—N2A—C14A—O1A	−18.3 (13)
N2—N1—C8—C7	165.6 (6)	N1A—N2A—C14A—C15A	22.1 (11)
C1—N1—C8—C7	25.1 (8)	C10A—N2A—C14A—C15A	165.3 (7)
C6—C7—C8—C9	−145.6 (7)	O1A—C14A—C15A—C16A	−139.4 (8)
C6—C7—C8—N1	−22.9 (7)	N2A—C14A—C15A—C16A	37.2 (10)
C14—N2—C10—C11	54.6 (11)	O1A—C14A—C15A—C20A	39.4 (11)
N1—N2—C10—C11	−127.3 (7)	N2A—C14A—C15A—C20A	−144.1 (6)
C12—O2—C11—O3	1.3 (12)	C20A—C15A—C16A—C17A	0.0
C12—O2—C11—C10	166.1 (8)	C14A—C15A—C16A—C17A	178.7 (7)
N2—C10—C11—O3	−156.4 (8)	C15A—C16A—C17A—C18A	0.0
N2—C10—C11—O2	40.0 (10)	C15A—C16A—C17A—S1	176.6 (6)
C11—O2—C12—C13	−164.7 (8)	O5—S1—C17A—C16A	133.4 (4)
N1—N2—C14—O1	−162.5 (7)	O4—S1—C17A—C16A	6.0 (5)
C10—N2—C14—O1	15.7 (11)	N3—S1—C17A—C16A	−111.6 (4)
N1—N2—C14—C15	24.8 (10)	O5—S1—C17A—C18A	−50.2 (5)
C10—N2—C14—C15	−157.0 (7)	O4—S1—C17A—C18A	−177.6 (4)
O1—C14—C15—C16	−143.7 (6)	N3—S1—C17A—C18A	64.8 (5)
N2—C14—C15—C16	29.1 (9)	C16A—C17A—C18A—C19A	0.0
O1—C14—C15—C20	35.0 (9)	S1—C17A—C18A—C19A	−176.2 (6)
N2—C14—C15—C20	−152.3 (6)	C16A—C17A—C18A—Cl1A	179.1 (10)
C20—C15—C16—C17	0.0	S1—C17A—C18A—Cl1A	2.9 (10)
C14—C15—C16—C17	178.6 (6)	C17A—C18A—C19A—C20A	0.0
C15—C16—C17—C18	0.0	Cl1A—C18A—C19A—C20A	−179.2 (10)
C15—C16—C17—S1	−179.3 (5)	C18A—C19A—C20A—C15A	0.0
O5—S1—C17—C16	134.0 (3)	C16A—C15A—C20A—C19A	0.0
O4—S1—C17—C16	2.2 (4)	C14A—C15A—C20A—C19A	−178.7 (7)
N3—S1—C17—C16	−111.6 (3)	C25—N3—C21—C22	78.8 (4)
O5—S1—C17—C18	−45.2 (4)	S1—N3—C21—C22	−105.9 (4)
O4—S1—C17—C18	−177.1 (3)	C23A—O7—C22—O6	−5.5 (10)
N3—S1—C17—C18	69.2 (4)	C23—O7—C22—O6	9.4 (9)
C16—C17—C18—C19	0.0	C23A—O7—C22—C21	176.0 (9)
S1—C17—C18—C19	179.2 (5)	C23—O7—C22—C21	−169.2 (8)
C16—C17—C18—Cl1	179.8 (8)	N3—C21—C22—O6	−179.2 (4)
S1—C17—C18—Cl1	−1.0 (8)	N3—C21—C22—O7	−0.5 (5)
C17—C18—C19—C20	0.0	C22—O7—C23—C24	177.1 (10)
Cl1—C18—C19—C20	−179.8 (8)	C22—O7—C23A—C24A	131.8 (11)
C18—C19—C20—C15	0.0	C21—N3—C25—C26	74.6 (4)
C16—C15—C20—C19	0.0	S1—N3—C25—C26	−100.5 (3)
C14—C15—C20—C19	−178.7 (6)	C27—O9—C26—O8	4.1 (7)
C1A—N1A—N2A—C14A	−135.0 (9)	C27—O9—C26—C25	−173.5 (4)
C8A—N1A—N2A—C14A	103.1 (8)	N3—C25—C26—O8	5.9 (6)
C1A—N1A—N2A—C10A	79.2 (10)	N3—C25—C26—O9	−176.5 (3)
C8A—N1A—N2A—C10A	−42.7 (8)	C26—O9—C27—C28	168.9 (4)
N2A—N1A—C1A—C2A	35 (2)		

### Hydrogen-bond geometry (Å, º)

*Cg*4 is the centroid of the major orientation of the C15–C20 benzene ring.

**Table:**

*D*—H···*A*	*D*—H	H···*A*	*D*···*A*	*D*—H···*A*
C2—H2···O2	0.95	2.37	3.198 (13)	145
C4—H4···O9^i^	0.95	2.48	3.262 (8)	139
C10—H10*A*···Cl1^i^	0.99	2.88	3.708 (11)	141
C24—H24*A*···Cl1	0.98	2.86	3.51 (2)	124
C25—H25*A*···O5^ii^	0.99	2.41	3.100 (5)	126
C27—H27*B*···*Cg*4^ii^	0.99	2.92	3.742 (7)	141

Symmetry codes: (i) *x*+1, *y*, *z*; (ii) −*x*+1, *y*−1/2, −*z*+1.
